# Social Risks and Nonadherence to Recommended Cancer Screening Among US Adults

**DOI:** 10.1001/jamanetworkopen.2024.49556

**Published:** 2025-01-03

**Authors:** Ami E. Sedani, Scarlett L. Gomez, Wayne R. Lawrence, Justin X. Moore, Heather M. Brandt, Charles R. Rogers

**Affiliations:** 1 Department of Epidemiology, School of Public Health, University of Texas Health Sciences Center at Houston, Dallas; 2 Department of Epidemiology and Biostatistics, University of California, San Francisco; 3 Division of Cancer Epidemiology and Genetics, National Cancer Institute, Rockville, Maryland; 4 Center for Health Equity Transformation, Markey Cancer Center, University of Kentucky College of Medicine, Lexington; 5 Epidemiology & Cancer Control, St Jude Children’s Research Hospital, Memphis, Tennessee; 6 Men’s Health Inequities Research Lab, Milwaukee, Wisconsin

## Abstract

**Importance:**

Research indicates that social drivers of health are associated with cancer screening adherence, although the exact magnitude of these associations remains unclear.

**Objective:**

To investigate the associations between individual-level social risks and nonadherence to guideline-recommended cancer screenings.

**Design, Setting, and Participants:**

This cross-sectional study used 2022 Behavioral Risk Factor Surveillance System data from 39 US states and Washington, DC. Analyses for each specific cancer screening subsample were limited to screening-eligible participants according to the latest US Preventive Services Task Force (USPSTF) guidelines. Data were analyzed from February 22 to June 5, 2024.

**Exposures:**

Ten social risk items, including life satisfaction, social and emotional support, social isolation, employment stability, food security (2 questions), housing security, utility security, transportation access, and mental well-being.

**Main Outcomes and Measures:**

Up-to-date status (adherence) was assessed using USPSTF definitions. Adjusted risk ratios (ARRs) and 95% CIs were estimated using modified Poisson regression with robust variance estimator.

**Results:**

A total of 147 922 individuals, representing a weighted sample of 78 784 149 US adults, were included in the analysis (65.8% women; mean [SD] age, 56.1 [13.3] years). The subsamples included 119 113 individuals eligible for colorectal cancer screening (CRCS), 7398 eligible for lung cancer screening (LCS), 56 585 eligible for cervical cancer screening (CCS), and 54 506 eligible for breast cancer screening (BCS). Findings revealed slight differences in effect size magnitude and in some cases direction; therefore results were stratified by sex, although precision was reduced for LCS. For the social contextual variables, life dissatisfaction was associated with nonadherence for CCS (ARR, 1.08; 95% CI, 1.01-1.16) and BCS (ARR, 1.22; 95% CI, 1.15-1.29). Lack of support was associated with nonadherence in CRCS in men and women and BCS, as was feeling isolated in CRCS in women and BCS. An association with feeling mentally distressed was seen in BCS. Under economic stability, food insecurity was associated with increased risk of nonadherence in CRCS in both men and women, CCS, and BCS; the direction of effect sizes for LCS were the same, but were not statistically significant. Under built environment, transportation insecurity was associated with nonadherence in CRCS in women and BCS, and cost barriers to health care access were associated with increased risk of nonadherence in CRCS for both men and women, LCS in women, and BCS, with the greatest risk and with reduced precision seen in LCS in women (ARR, 1.54; 95% CI, 1.01-2.33).

**Conclusions and Relevance:**

In this cross-sectional study of adults eligible for cancer screening, findings revealed notable variations in screening patterns by both screening type and sex. Given that these risks may not always align with patient-centered social needs, further research focusing on specific target populations is essential before effective interventions can be implemented.

## Key Points

**Question:**

How are social risks associated with nonadherence to the US Preventive Services Task Force cancer screening guidelines?

**Findings:**

This cross-sectional study of 147 922 screening-eligible adults (weighted to represent 78 784 149 US adults) found that social risks were independently and differentially associated with nonadherence to cancer screenings, with some of these associations varying by sex.

**Meaning:**

These results highlight the importance of assessing and addressing social risks to improve adherence to recommended cancer screening; further research is essential before effective interventions can be implemented, as social risks may not always align with patient-centered social needs.

## Teaser

This cross-sectional study investigates the associations between individual-level social risks and nonadherence to guideline-recommended screening for colorectal, lung, breast, and cervical cancers in a weighted sample of US adults.

## Introduction

Routine cancer screenings are vital for early detection and prevention of more invasive cancers, which improves treatment outcomes and reduces mortality rates significantly. For individuals at average risk, routine cancer screenings facilitate the identification of preclinical cancer or precancerous lesions. In some cases, they enable timely interventions, reducing the risk of advanced-stage diagnoses, which are associated with poorer clinical outcomes. Despite endorsements from multiple advisory bodies for colorectal cancer screening (CRCS), lung cancer screening (LCS), breast cancer screening (BCS), and cervical cancer screening (CCS), the rates among eligible adults in the US remain below recommended targets.^1^ Furthermore, significant disparities in evidence-based screening adherence persist, disproportionately affecting structurally marginalized populations.^2,3^ These disparities, rooted in a complex interplay of factors exacerbated by longstanding structural inequities and societal injustices, pose a substantial challenge to public health and impede progress toward achieving health equity.^4^

To address these challenges, researchers have developed various frameworks to understand and mitigate the factors influencing health outcomes and disparities, particularly in cancer. Proposed frameworks encompass a range of interrelated factors known as social drivers of health (SDOH), which shape individuals’ social risks (eg, food insecurity, housing instability) and needs.^5,6,7^ Recognizing that SDOH serve as pivotal upstream factors driving these disparities, the cancer research community has begun assessing and addressing SDOH across the cancer control continuum.^2,8,9,10,11,12,13^ While notable progress has been made, critical gaps remain in identifying the specific drivers that contribute to nonadherence to screening recommendations. Several existing studies have relied on health care–based samples, which may limit our understanding of the broader landscape of cancer screening adherence.^14,15,16,17,18^ Recent efforts using nationally representative population-based samples have sought to fill this void. Although useful, these studies relied on pre–COVID-19 pandemic data, used incomplete definitions of screening adherence, and/or focused on only a single type of screening.^19,20,21,22^ This study aims to fill these critical gaps by providing the most current epidemiological data on nonadherence across 4 cancer screening types, exploring effect modified by sex, and examining individual social risks without conflating them with other SDOH. Strengthening epidemiological evidence regarding these associations is essential for informing targeted public health interventions as well as identifying specific directions of future research.

The primary objective of this study was to investigate the associations between individual-level social risks and nonadherence to guideline-recommended screenings for colorectal, lung, breast, and cervical cancers, using data from the Behavioral Risk Factor Surveillance System (BRFSS). Additionally, we explored whether sex was associated with nonadherence for CRCS and LCS. An enhanced understanding is crucial for advancing health equity and defining practical strategies and specific focal points for targeted interventions.

## Methods

### Data Source, Study Design, and Study Samples

Consistent with the Common Rule, this cross-sectional study of deidentified publicly available BRFSS data did not require institutional review board approval or informed consent. Results reporting is based on the Strengthening the Reporting of Observational Studies in Epidemiology (STROBE) and American Association for Public Opinion Research (AAPOR) reporting guidelines.

The BRFSS is an annual, nationally representative, cross-sectional, random-digit–dialed telephone survey overseen by the Centers for Disease Control and Prevention (CDC) that collects data on health-related risk behaviors and preventive health practices among noninstitutionalized US adults (aged ≥18 years).^23^ Cancer screening questions are part of the BRFSS rotating core component in even-numbered years. The Social Determinants of Health and Health Equity (SDOH-HE) module was introduced as an optional module in 2022. It was deployed in 39 states and Washington, DC, covering approximately 77% of the US adult population (eFigure 1 in Supplement 1).^24^ The median survey response rate for all states, territories, and Washington, DC, in 2022, using the AAPOR R4 definition, was 45.1% and ranged from 22.8% to 66.8%.^25^

To create our analytical study populations, we first identified respondents in states that administered the 2022 SDOH-HE module. We excluded respondents from the 2 participating US territories (Puerto Rico and the US Virgin Islands) due to concerns about differences in external factors influencing the exposure and outcome. The analytical populations were then restricted to participants eligible for screening based on US Preventive Services Task Force (USPSTF) recommendations. The ascertainment process for each specific cancer subsample is available in eFigure 2 in Supplement 1.

### Measures

The primary outcome assessed was whether participants were adherent with USPSTF-recommended cancer screening guidelines as of 2022 (eTable 1 in Supplement 1). Respondents were asked whether they had ever had a specific screening test and when their most recent test occurred. Social risks were based on items from the SDOH-HE module comprised of 10 questions assessing life satisfaction, social and emotional support, social isolation, employment stability, food security (2 questions), housing security, utility security, transportation access, and mental well-being.^26,27,28^ For consistency, we used the CDC’s suggested recoding from participants’ original response options to each question (eTable 2 in Supplement 1).^29^ We also included a variable for cost as a barrier to health care access.

Based on existing literature, we included several covariates a priori to control for factors associated with social risks and cancer screening: self-reported sex, age, self-identified race and ethnicity, educational attainment, health insurance coverage, marital status, US Census region, urban-rural residence, and having a personal health care professional. We did not include annual household income due to a high proportion of missing data (24.2%).

### Statistical Analysis

Data analysis was performed from February 22 to June 5, 2024, using SAS, version 9.4 (SAS Institute Inc). To be nationally representative and account for nonresponse, all estimates used CDC-recommended weights. Given limited missing data (<10% for most covariates), we used a complete-case approach, excluding observations with missing values in any variables of interest (eTable 3 in Supplement 1). Descriptive statistics were generated to examine the characteristics of the 4 study cohorts (ie, participants eligible for CRCS, LCS, CCS, and BCS). Frequencies and weighted percentages with corresponding 95% CIs were calculated. To understand the associations between social risks and screening nonadherence, prevalence of nonadherence to each cancer screening type was examined by demographic variable, and unadjusted risk ratios (RRs) and 95% CIs were calculated. Next, to facilitate the identification of targets for effective interventions recommendations, we explored the associations between social risks and screening nonadherence. The RR and corresponding 95% CIs were estimated using modified Poisson regression with robust variance estimator (RR > 1.00 indicating nonadherence, and RR < 1.00 indicating adherence). CRCS and LCS models were stratified by sex (eTables 6 and 7 in Supplement 1). Because odds ratios are most commonly reported in the literature, we used weighted logistic regression to estimate odds ratios for comparability (eTables 5 in Supplement 1). Two-sided *P* < .05 was considered statistically significant.

## Results

### Sample Characteristics

An unweighted sample of 147 922 individuals (representing a weighted sample of 78 784 149 individuals) was analyzed. Unweighted subsamples for cancer screening type included 119 113 individuals (55 628 721 weighted) for CRCS, 7398 (3 462 205 weighted) for LCS, 56 585 (37 739 506 weighted) for CCS, and 54 506 (23 990 783 weighted) for BCS. The characteristics of the 4 study cohorts aligned with expectations, reflecting the study’s weighting methods and population-based design (Table 1). Given the unique eligibility criteria for LCS, the sample exhibited distinct demographic characteristics. Similar demographic patterns were observed for persons eligible for CCS and BCS, except for patterns of health insurance coverage and having a personal health care professional. Overall, 65.4% (95% CI, 64.8%-66.0%) of eligible participants were adherent with CRCS, 16.2% (95% CI, 14.7%-17.7%) with LCS, 51.6% (95% CI, 50.7%-52.4%) with CCS, and 77.7% (95% CI, 76.9%-78.5%) with BCS. Prevalences of social risks by screening cohort are shown in eTable 4 in Supplement 1.

**Table 1.** Characteristics of Cancer Screening–Eligible BRFSS Participants in 39 States and Washington, DC, 2022^a^

Characteristic	Eligible respondents, No. (weighted %) [95% CI]			
	CRCS (n = 119 113)	LCS (n = 7398)	CCS (n = 56 585)	BCS (n = 54 506)
Guideline adherence	82 636 (65.4) [64.8-66.0]	1365 (16.2) [14.7-17.7]	31 877 (51.6) [50.7-52.4]	42 404 (77.7) [76.9-78.5]
Sex				
Men	54 935 (48.1) [47.5-48.7]	3678 (51.4) [49.3-53.4]	NA	NA
Women	64 178 (51.9) [51.3-52.5]	3720 (48.6) [46.6-50.7]	NA	NA
Age group, y				
18-24	NA	NA	3236 (9.9) [9.3-10.5]	NA
25-34	NA	NA	10 741 (25.6) [24.9-26.4]	NA
35-44	NA	NA	13 463 (25.2) [24.4-25.9]	NA
45-54	32 106 (33.2) [32.6-33.8]	1192 (19.2) [17.5-20.9]	12 838 (19.1) [18.5-19.8]	9162 (21.3) [20.5-22.1]
55-64	40 427 (36.3) [35.7-36.9]	3045 (45.9) [43.8-48.0]	14 577 (18.4) [17.8-19.1]	21 741 (43.4) [42.5-44.4]
≥65	46 580 (30.4) [29.9-31.0]	3161 (34.9) [33.0-36.9]	1730 (1.7) [1.5-1.8]	23 603 (35.2) [34.4-36.1]
Race and ethnicity				
Hispanic	7144 (13.2) [12.7-13.8]	319 (8.2) [7.0-9.5]	6637 (21.1) [20.3-21.9]	2994 (12.2) [11.4-13.0]
Non-Hispanic American Indian or Alaska Native	1675 (1.3) [1.1-1.4]	181 (2.4) [1.7-3.0]	933 (1.2) [1.1-1.4]	759 (1.2) [1.0-1.4]
Non-Hispanic Asian, Native Hawaiian or Other Pacific Islander	1824 (5.0) [4.5-5.4]	30 (1.4) [0.3-2.5]	1670 (7.0) [6.4-7.6]	604 (4.6) [3.9-5.3]
Non-Hispanic Black	9064 (11.7) [11.3-12.1]	740 (13.7) [12.3-15.1]	5019 (12.5) [12.0-13.0]	4687 (12.0) [11.5-12.7]
Non-Hispanic White	97 673 (66.3) [65.6-66.9]	5965 (70.9) [68.9-72.9]	41 089 (54.8) [54.0-55.6]	44 760 (67.5) [66.5-68.5]
Non-Hispanic multiracial	1733 (2.5) [2.3-2.8]	163 (3.5) [2.7-4.3]	1237 (3.4) [3.0-3.7]	702 (2.4) [2.0-2.7]
Educational attainment				
Less than high school	5558 (10.2) [9.7-10.7]	852 (19.5) [17.6-21.4]	2719 (10.4) [9.7-11.2]	2351 (9.3) [8.6-10.0]
High school diploma or GED	26 113 (24.0) [23.5-24.6]	2673 (34.8) [32.9-36.7]	10 334 (21.5) [20.8-22.2]	11 577 (23.6) [22.9-24.4]
Some college	33 060 (31.7) [31.1-32.4]	2463 (33.0) [31.1-34.9]	15 012 (31.4) [30.6-32.2]	16 137 (33.8) [32.9-34.7]
College degree or more^b^	54 382 (34.0) [33.5-34.6]	1410 (12.6) [11.2-14.1]	28 520 (36.7) [35.9-37.4]	24 441 (33.3) [32.4-34.1]
Marital status				
Married	73 011 (63.9) [63.32-64.5]	2803 (41.7) [39.5-43.8]	31 014 (52.5) [51.7-53.4]	31 304 (60.1) [59.2-61.0]
Divorced, separated, or widowed	32 325 (24.4) [23.9-24.9]	3605 (42.3) [40.3-44.2]	10 498 (15.6) [15.0-16.2]	17 652 (29.9) [29.1-30.8]
Never married	13 777 (11.7) [11.3-12.1]	990 (16.1) [14.3-17.8]	15 073 (31.9) [31.1-32.7]	5550 (10.0) [9.4-10.6]
Area of residence				
Urban	101 441 (92.4) [92.2-92.6]	6143 (90) [89.2-90.9]	49 961 (94.1) [93.9-94.4]	46 169 (92.4) [92.1-92.7]
Rural	17 672 (7.6) [7.3-7.8]	1255 (10) [9.1-10.8]	6624 (5.8) [5.6-6.1]	8337 (7.6) [7.3-7.9]
US Census region				
Northeast	22 696 (10.5) [10.3-10.7]	1184 (9.1) [8.4-9.7]	11 164 (10.7) [10.5-10.9]	10 558 (10.7) [10.4-11.0]
Midwest	32 161 (23.0) [22.8-23.2]	2193 (27.6) [26.7-28.5]	14 725 (22.2) [21.9-22.5]	14 521 (22.7) [22.4-23.1]
South	32 910 (41.7) [41.3-42.1]	2464 (47.5) [46.3-48.8]	15 462 (40.1) [39.6-40.6]	15 556 (42.2) [41.6-42.7]
West	31 346 (24.8) [24.3-25.2]	1557 (15.8) [14.6-17.1]	15 234 (27.0) [26.4-27.6]	13 871 (24.4) [23.7-25.1]
Health insurance coverage				
Private	56 956 (51.9) [51.3-52.5]	2002 (29.2) [27.4-31.1]	37 605 (63.0) [62.2-63.8]	7009 (32.5) [31.3-33.7]
Public	57 627 (42.7) [42.1-43.3]	5056 (65.3) [63.3-67.2]	15 114 (27.9) [27.1-28.6]	16 966 (63.7) [62.5-64.9]
Uninsured	4530 (5.4) [5.1-5.7]	340 (5.5) [4.6-6.4]	3866 (9.2) [8.6-9.7]	652 (3.8) [3.2-4.4]
Personal health care professional				
No	9695 (9.5) [9.1-9.9]	632 (9.5) [8.3-10.7]	7365 (16.4) [15.7-17.1]	1388 (6.8) [6.0-7.5]
Yes	109 418 (90.5) [90.1-90.9]	6766 (90.5) [89.3-91.7]	49 220 (83.6) [82.9-84.3]	23 239 (93.2) [92.5-94.0]

Abbreviations: BCS, breast cancer screening; BRFSS, Behavioral Risk Factor Surveillance System; CCS, cervical cancer screening; CRCS, colorectal cancer screening; GED, General Educational Development certification; LCS, lung cancer screening; NA, not applicable.

^a^ Data available for 39 states (Alabama, Alaska, Arizona, California, Connecticut, Delaware, Florida, Georgia, Idaho, Indiana, Iowa, Kansas, Kentucky, Maine, Maryland, Massachusetts, Michigan, Minnesota, Mississippi, Missouri, Montana, Nebraska, Nevada, New Hampshire, New Jersey, New Mexico, North Carolina, Ohio, Oklahoma, Rhode Island, South Carolina, Tennessee, Texas, Utah, Vermont, Washington, West Virginia, Wisconsin, and Wyoming) and Washington, DC.

^b^ Includes completion of technical school.

The prevalence of nonadherence by participant characteristics is shown in Table 2. For CRCS, LCS, and BCS, the prevalence of nonadherence decreased with age; however, for CCS, nonadherence was lowest for the group aged 45 to 54 years. The prevalence of nonadherence decreased as educational attainment increased for all screening tests except for LCS. For CRCS and LCS, nonadherence was lower among individuals with public health insurance coverage than among those with private insurance. The prevalence of nonadherence was higher for participants experiencing social risks for all screening tests.

**Table 2.** Prevalence and Risk Ratios of Screening Nonadherence by Cancer Screening Test, BRFSS, 2022

Variable	CRCS (n = 119 113)		LCS (n = 7398)		CCS (n = 56 585)		BCS (n = 54 506)	
	Prevalence, No. (weighted %)	RR (95% CI)	Prevalence, No. (weighted %)	RR (95% CI)	Prevalence, No. (weighted %)	RR (95% CI)	Prevalence, No. (weighted %)	RR (95% CI)
**Guideline nonadherence**	36 477 (34.6)	NA	6033 (83.8)	NA	22 150 (44.2)	NA	12 102 (22.3)	NA
**Demographics**								
Sex								
Men	17 380 (35.9)	1.08 (1.04-1.12)	2959 (82.6)	0.97 (0.94-1.01)	NA	NA	NA	NA
Women	19 097 (33.4)	1 [Reference]	3074 (85.0)	1 [Reference]	NA	NA	NA	NA
Age group, y								
21-24	NA	NA	NA	NA	2154 (71.5)	1.63 (1.47-1.81)	NA	NA
25-34	NA	NA	NA	NA	4948 (48.4)	1.10 (0.99-1.23)	NA	NA
35-44	NA	NA	NA	NA	4860 (39.8)	0.91 (0.81-1.01)	NA	NA
45-54	18 135 (57.8)	3.11 (2.97-3.25)	1111 (92.9)	1.21 (1.16-1.26)	4101 (36.1)	0.82 (0.73-0.93)	2331 (25.0)	1.32 (1.20-1.44)
55-64	10 015 (26.7)	1.44 (1.36-1.52)	2549 (85.2)	1.11 (1.06-1.16)	5361 (38.3)	0.87 (0.78-0.98)	5154 (23.6)	1.24 (1.15-1.34)
≥65	8327 (18.6)	1 [Reference]	2373 (77.0)	1 [Reference]	726 (43.8)	1 [Reference]	4617 (19.0)	1 [Reference]
Race and ethnicity								
Hispanic	2169 (42.1)	1.35 (1.26-1.46)	318 (85.6)	1.06 (0.93-1.20)	1953 (52.9)	1.40 (1.31-1.50)	648 (28.2)	1.26 (1.07-1.50)
Non-Hispanic Black	2649 (33.2)	1.07 (1.00-1.13)	673 (92.3)	1.14 (1.10-1.18)	2280 (47.4)	1.26 (1.19-1.33)	740 (16.9)	0.76 (0.67-0.86)
Non-Hispanic White	28 384 (31.1)	1 [Reference]	4756 (81.2)	1 [Reference]	14 493 (37.6)	1 [Reference]	9964 (22.3)	1 [Reference]
Non-Hispanic other^a^	3275 (48.4)	1.56 (1.47-1.64)	286 (90.7)	1.12 (1.05-1.19)	3424 (54.8)	1.46 (1.39-1.53)	750 (24.0)	1.08 (0.94-1.23)
Educational attainment								
Less than high school	2735 (53.6)	1.81 (1.71-1.91)	712 (85.0)	1.02 (0.94-1.12)	1711 (64.7)	1.94 (1.82-2.07)	773 (34.0)	1.93 (1.70-2.18)
High school diploma or GED	9056 (36.0)	1.21 (1.16-1.27)	2174 (83.0)	1.00 (0.93-1.08)	5345 (54.3)	1.63 (1.55-1.71)	2986 (25.7)	1.45 (1.33-1.59)
Some college	10 052 (32.7)	1.10 (1.05-1.15)	1999 (84.2)	1.01 (0.94-1.10)	6112 (43.3)	1.30 (1.24-1.36)	3800 (21.3)	1.21 (1.10-1.32)
College degree^b^	14 634 (29.7)	1 [Reference]	1148 (83.0)	1 [Reference]	8982 (33.3)	1 [Reference]	4543 (17.7)	1 [Reference]
Marital status								
Married	20 365 (31.7)	1 [Reference]	2238 (81.9)	1 [Reference]	10 699 (38.8)	1 [Reference]	5959 (19.3)	1 [Reference]
Divorced, separated, or widowed	10 427 (35.9)	1.13 (1.09-1.18)	2970 (83.7)	1.02 (0.98-1.07)	4144 (42.2)	1.09 (1.03-1.15)	4661 (27.0)	1.39 (1.30-1.50)
Never married	5685 (47.4)	1.49 (1.43-1.56)	825 (89.0)	1.09 (1.04-1.14)	7307 (54.3)	1.40 (1.34-1.46)	1482 (26.1)	1.35 (1.22-1.50)
Area of residence								
Urban	30 639 (34.5)	1 [Reference]	5036 (83.9)	1 [Reference]	19 433 (44.1)	1 [Reference]	9997 (22.0)	1 [Reference]
Rural	5838 (36.3)	1.06 (1.01-1.10)	997 (83.3)	0.99 (0.95-1.04)	2717 (47.1)	1.07 (1.01-1.13)	2105 (25.9)	1.18 (1.08-1.28)
US Census region								
Northeast	5981 (31.1)	1 [Reference]	915 (76.9)	1 [Reference]	3862 (40.9)	1 [Reference]	1905 (18.6)	1 [Reference]
Midwest	9625 (32.4)	1.04 (0.99-1.09)	1745 (80.3)	1.04 (0.97-1.12)	5659 (41.4)	1.01 (0.96-1.06)	3026 (22.1)	1.19 (1.07-1.31)
South	10 137 (34.5)	1.11 (1.06-1.16)	2046 (86.3)	1.12 (1.03-1.23)	6313 (45.0)	1.10 (1.05-1.16)	3406 (22.5)	1.21 (1.09-1.34)
West	10 734 (38.3)	1.23 (1.16-1.30)	1327 (86.4)	1.12 (1.03-1.23)	6316 (46.7)	1.14 (1.08-1.21)	3765 (23.9)	1.29 (1.14-1.45)
Health insurance coverage								
Private	19 504 (36.7)	1 [Reference]	1713 (87.1)	1 [Reference]	12 709 (38.1)	1 [Reference]	4941 (19.4)	1 [Reference]
Public	13 645 (26.9)	0.73 (0.70-0.76)	3996 (81.4)	0.94 (0.90-0.97)	7044 (51.1)	1.34 (1.29-1.40)	6146 (22.3)	1.15 (1.06-1.23)
Uninsured	3328 (75.5)	2.06 (1.97-2.15)	324 (94.5)	1.08 (1.05-1.13)	2397 (65.3)	1.71 (1.63-1.80)	1015 (60.5)	3.11 (2.84-3.41)
**Social risks**								
Life dissatisfaction								
No	34 175 (34.2)	1 [Reference]	5326 (83.2)	1 [Reference]	20 729 (43.6)	1 [Reference]	11 030 (21.4)	1 [Reference]
Yes	2302 (42.7)	1.25 (1.17-1.33)	707 (88.1)	1.06 (1.01-1.11)	1421 (54.0)	1.24 (1.16-1.32)	1072 (38.8)	1.81 (1.63-2.01)
Lack of support								
No	27 622 (32.6)	1 [Reference]	4105 (83.5)	1 [Reference]	16 767 (42.4)	1 [Reference]	9068 (20.4)	1 [Reference]
Yes	8855 (42.2)	1.30 (1.24-1.35)	1928 (84.4)	1.01 (0.97-1.05)	5383 (50.3)	1.18 (1.13-1.24)	3034 (30.0)	1.47 (1.36-1.60)
Feeling isolated								
No	25 599 (33.1)	1 [Reference]	3777 (83.9)	1 [Reference]	14 341 (42.1)	1 [Reference]	7838 (20.0)	1 [Reference]
Yes	10 878 (38.7)	1.17 (1.13-1.21)	2256 (83.7)	1.00 (0.96-1.03)	7809 (48.5)	1.15 (1.11-1.20)	4264 (28.3)	1.41 (1.32-1.52)
Mentally distressed								
No	32 103 (33.7)	1 [Reference]	4869 (83.0)	1 [Reference]	18 285 (43.8)	1 [Reference]	10 306 (21.3)	1 [Reference]
Yes	4374 (42.5)	1.26 (1.20-1.32)	1164 (87.5)	1.05 (1.02-1.10)	3865 (46.2)	1.05 (1.00-1.11)	1796 (30.5)	1.43 (1.31-1.56)
Employment insecurity								
No	32 620 (33.2)	1 [Reference]	5460 (83.2)	1 [Reference]	18 993 (43.3)	1 [Reference]	10 907 (21.6)	1 [Reference]
Yes	3857 (47.8)	1.44 (1.36-1.52)	573 (88.7)	1.07 (1.02-1.11)	3157 (49.6)	1.14 (1.08-1.21)	1195 (30.4)	1.41 (1.25-1.58)
Receiving SNAP								
No	32 352 (33.4)	1 [Reference]	4504 (83.0)	1 [Reference]	18 261 (42.6)	1 [Reference]	10 429 (20.9)	1 [Reference]
Yes	4125 (45.8)	1.37 (1.30-1.44)	1529 (85.9)	1.03 (0.99-1.08)	3889 (52.1)	1.22 (1.17-1.28)	1673 (33.3)	1.59 (1.45-1.75)
Food insecurity								
No	31 841 (32.9)	1 [Reference]	4582 (82.6)	1 [Reference]	18 262 (41.9)	1 [Reference]	10 269 (20.5)	1 [Reference]
Yes	4636 (47.1)	1.43 (1.37-1.50)	1451 (87.1)	1.05 (1.02-1.09)	3888 (56.2)	1.34 (1.28-1.40)	1833 (34.1)	1.67 (1.52-1.82)
Housing insecurity								
No	32 289 (33.1)	1 [Reference]	4880 (82.4)	1 [Reference]	18 525 (43.0)	1 [Reference]	10 539 (20.9)	1 [Reference]
Yes	4188 (49.6)	1.50 (1.43-1.57)	1153 (89.6)	1.09 (1.05-1.13)	3625 (50.8)	1.18 (1.12-1.24)	1563 (35.7)	1.71 (1.55-1.88)
Utility insecurity								
No	33 568 (33.6)	1 [Reference]	5251 (82.9)	1 [Reference]	19 874 (43.8)	1 [Reference]	10 981 (21.2)	1 [Reference]
Yes	2909 (49.5)	1.47 (1.39-1.56)	782 (89.1)	1.07 (1.03-1.12)	2276 (48.1)	1.10 (1.04-1.17)	1121 (37.7)	1.78 (1.59-1.99)
Transportation insecurity								
No	33 772 (33.7)	1 [Reference]	5152 (83.5)	1 [Reference]	19 906 (43.1)	1 [Reference]	10 903 (21.2)	1 [Reference]
Yes	2705 (49.2)	1.46 (1.38-1.54)	881 (85.6)	1.03 (0.97-1.08)	2244 (56.3)	1.31 (1.24-1.38)	1199 (40.5)	1.92 (1.74-2.11)
Cost as a barrier to health care								
No	32 523 (32.8)	1 [Reference]	5217 (82.7)	1 [Reference]	18 803 (42.6)	1 [Reference]	10 541 (20.5)	1 [Reference]
Yes	3954 (55.2)	1.68 (1.61-1.76)	816 (90.7)	1.10 (1.06-1.14)	3347 (54.1)	1.27 (1.21-1.33)	1561 (43.8)	2.13 (1.96-2.32)
Cumulative social risks, mean (SD)^c^	1.4 (2.0)	1.07 (1.06-1.08)	2.2 (2.3)	1.09 (1.04-1.13)	1.8 (2.2)	1.05 (1.04-1.06)	1.7 (2.1)	1.06 (1.05-1.07)

Abbreviations: BCS, breast cancer screening; BRFSS, Behavioral Risk Factor Surveillance System; CCS, cervical cancer screening; CRCS, colorectal cancer screening; GED, General Educational Development certification; LCS, lung cancer screening; NA, not applicable; RR, crude risk ratio (ie, prevalence ratio); SNAP, Supplemental Nutrition Assistance Program.

^a^ Includes non-Hispanic American Indian or Alaska Native, Asian, Native Hawaiian or Other Pacific Islander, and multiracial.

^b^ Includes completion of technical school.

^c^ The mean for the summary measure represents the cumulative number of adverse social risks each individual experienced, calculated by assigning a value of 1 for each social risk present and 0 for each risk absent. We then summed these binary values across all social risks included in the study for each individual, providing a total count of risks per person. The mean was then derived by averaging these total risk counts across each subsample.

### Adverse Social Risks and Cancer Screening Nonadherence

The adjusted RRs (ARRs) for dissatisfaction showed a consistent trend above 1.00 for all subgroups, although not all results were statistically significant (Table 3). In a number of cases, even though ARRs were higher, the results were not statistically significant. The ARR of BCS nonadherence was 1.22 (95% CI, 1.15-1.29) for individuals who reported feeling dissatisfied or very dissatisfied with life compared with those who reported feeling satisfied or very satisfied. CCS was also associated with greater nonadherence (ARR, 1.08; 95% CI, 1.01-1.16), but although the ARRs were higher for both men and women in CRCS (ARR, 1.05 [95% CI, 0.99-1.12] for men and 1.04 [95% CI, 0.76-1.83] for women) and LCS (ARR, 1.18 [95% CI, 0.88-1.89] for men and 1.29 [95% CI, 0.88-1.89] for women), those results did not reach statistical significance. Lack of social and emotional support was associated with nonadherence for all CRC in both sexes and BCS; the ARRs for LCS in women and CCS were higher, although these results were not significant. An inverse association was also observed for feeling isolated and LCS, regardless of sex. Feeling mentally distressed was associated with BCS adherence (ARR, 1.08; 95% CI, 1.04-1.12).

**Table 3.** Adjusted Risk Ratios of Cancer Screening Nonadherence and Social Risks, BRFSS, 2022

Social risk	ARR (95% CI)^a^					
	Colorectal		Lung		Cervical (n = 56 585)	Breast (n = 54 506)
	Men (n = 54 935)	Women (n = 64 178)	Men (n = 3678)	Women (n = 3720)		
Social context						
Life dissatisfaction	1.05 (0.99-1.12)	1.04 (0.98-1.10)	1.18 (0.76-1.83)	1.29 (0.88-1.89)	1.08 (1.01-1.16)	1.22 (1.15-1.29)
Lack of support	1.08 (1.04-1.12)	1.04 (1.01-1.08)	0.84 (0.61-1.16)	1.14 (0.87-1.49)	1.03 (0.99-1.07)	1.08 (1.05-1.12)
Feeling isolated	1.04 (1.01-1.07)	1.03 (1.00-1.05)	0.96 (0.72-1.29)	0.88 (0.70-1.12)	1.01 (0.98-1.05)	1.08 (1.05-1.10)
Mentally distressed	1.00 (0.95-1.05)	1.02 (0.98-1.06)	1.15 (0.78-1.68)	1.19 (0.89-1.60)	0.97 (0.93-1.00)	1.08 (1.04-1.12)
Total social score^b^	1.03 (1.01-1.05)	1.02 (1.00-1.03)	0.98 (0.87-1.11)	1.04 (0.94-1.14)	1.01 (0.99-1.02)	1.05 (1.04-1.06)
Economic stability						
Employment insecurity	1.15 (1.08-1.23)	1.07 (1.00-1.14)	0.91 (0.61-1.34)	1.55 (0.87-2.77)	0.96 (0.91-1.01)	1.06 (1.01-1.11)
Receiving SNAP	1.05 (0.99-1.12)	1.08 (1.02-1.14)	0.86 (0.62-1.20)	1.24 (0.90-1.71)	0.96 (0.91-1.02)	1.11 (1.06-1.16)
Food insecurity	1.11 (1.04-1.17)	1.09 (1.04-1.15)	1.18 (0.85-1.63)	1.12 (0.83-1.50)	1.06 (1.00-1.12)	1.12 (1.07-1.17)
Housing insecurity	1.17 (1.08-1.25)	1.04 (0.98-1.09)	1.19 (0.81-1.76)	1.46 (1.03-2.08)	0.95 (0.90-1.00)	1.14 (1.09-1.20)
Utility services insecurity	1.18 (1.08-1.29)	1.05 (0.99-1.12)	1.24 (0.80-1.92)	1.22 (0.84-1.77)	0.95 (0.90-1.00)	1.19 (1.12-1.27)
Total economic score^c^	1.06 (1.03-1.08)	1.04 (1.02-1.06)	1.03 (0.90-1.18)	1.13 (1.01-1.28)	0.99 (0.97-1.00)	1.06 (1.05-1.08)
Built environment						
Transportation insecurity	1.06 (0.98-1.14)	1.15 (1.08-1.22)	0.81 (0.53-1.26)	1.10 (0.75-1.60)	1.06 (0.99-1.14)	1.22 (1.15-1.30)
Health and health care						
Cost as barrier to care	1.13 (1.05-1.22)	1.15 (1.08-1.23)	1.29 (0.84-1.98)	1.54 (1.01-2.33)	1.03 (0.98-1.09)	1.25 (1.18-1.32)
Cumulative social risks^d^	1.03 (1.02-1.04)	1.02 (1.01-1.03)	1.00 (0.94-1.07)	1.06 (1.00-1.13)	1.00 (0.99-1.01)	1.04 (1.04-1.05)

Abbreviations: ARR, adjusted risk ratio; BRFSS, Behavioral Risk Factor Surveillance System; SNAP, Supplemental Nutrition Assistance Program.

^a^ Models were adjusted for age, race and ethnicity, educational attainment, health insurance coverage, marital status, and geography (census region), accounting for complex survey design (weight). *P* values can be found in eTable 8 in Supplement 1.

^b^ Scores ranged from 0 to 4, with higher scores indicating the individual faced a greater number of adverse social risks within the social and community context domain.

^c^ Scores ranged from 0 to 5, with higher scores indicating the individual faced a greater number of adverse social risks within the economic stability domain.

^d^ Ranged from 0 to 11, and risks are for every unit increase in number of social risks.

Employment insecurity was associated with nonadherence to CRCS in both men (ARR, 1.15; 95% CI, 1.08-1.23) and women (ARR, 1.07; 95% CI, 1.00-1.14) and for BCS (ARR, 1.06; 95% CI, 1.01-1.11). For LCS among women, a higher ARR was observed (1.55; 95% CI, 0.87-2.77), though not statistically significant. Conversely, lower ARRs were noted for nonadherence to LCS among men (0.91; 95% CI, 0.61-1.34) and CCS (0.96; 95% CI, 0.91-1.01), but neither result was statistically significant. Similar results were observed for receiving Supplemental Nutrition Assistance Program benefits. Food insecurity corresponded with higher ARRs for nonadherence across all 4 cancer screening tests. For CRCS, associations were observed among both men (ARR, 1.11; 95% CI, 1.04-1.17) and women (ARR, 1.09; 95% CI, 1.04-1.15), as well as for CCS (ARR, 1.06; 95% CI, 1.00-1.12) and BCS (ARR, 1.12; 95% CI, 1.07–1.17). For LCS, precision was reduced, resulting in wider 95% CIs, and although the ARRs were elevated, they did not reach statistical significance for either men (1.18; 95% CI, 0.85-1.63) or women (1.12; 95% CI, 0.83-1.50). Housing insecurity was associated with nonadherence to CRCS in men (ARR, 1.17; 95% CI, 1.08-1.25), LCS among women (ARR, 1.46; 95% CI, 1.03-2.08), and BCS (ARR, 1.14; 95% CI, 1.09-1.20). Conversely, a slight inverse association was observed for CCS (ARR, 0.95; 95% CI, 0.90-1.00). Higher ARRs were also observed for CRCS among women (1.04; 95% CI, 0.98-1.09) and LCS among men (1.19; 95% CI, 0.81-1.76), but neither result was statistically significant. Similar trends were observed for utility services insecurity.

Transportation insecurity corresponded with higher ARRs for nonadherence across cancer screening types, except for LCS among men. The largest effect sizes were observed for CRCS among women (ARR, 1.15; 95% CI, 1.08-1.22) and for BCS (ARR, 1.22; 95% CI, 1.15-1.32), both of which were statistically significant. In contrast, a lower ARR was observed for LCS among men (0.81; 95% CI, 0.53-1.26), though this result was not statistically significant. Cost as a barrier to health care access was associated with cancer screening nonadherence to CRC among men (ARR, 1.13; 95% CI, 1.05-1.22) and women (ARR, 1.15; 95% CI, 1.08-1.23), LCS among women (ARR, 1.54; 95% CI, 1.01- 2.33), and BCS (ARR, 1.25; 95% CI, 1.18-1.32). ARRs were also greater than 1.00 for LCS among men (1.29; 95% CI, 0.84-1.98) and CCS (1.03; 95% CI, 0.98-1.09), but did not reach statistical significance.

## Discussion

This cross-sectional study explored the association of individual-level social risks with adherence to USPSTF-recommended cancer screening in a sample of US adults. Our study had several key findings, including a differential and independent association between nonadherence to screenings and social risks such as cost as a barrier to health care access, transportation insecurity, and social context factors. We also found evidence of potential effect modification by sex. Furthermore, by analyzing individual variables contributing to composite scores, we found that overall measures often masked true effects. Examining variables separately provided clearer insights, aligning with prior research on the limitations of using combined indices.^30,31,32,33^

Our results indicate a consistent pattern where cost barriers to health care access corresponded with increased nonadherence to screenings across all 4 cancer types, though findings did not reach statistical significance for LCS among men and CCS. The lack of statistical significance for LCS among men may be attributable to smaller subsample sizes, while the marginal effect size for CCS may indicate that cost barriers are less pronounced in this subgroup, highlighting the need for further investigation to verify these trends and explore underlying factors. Nevertheless, these results suggest that cost barriers may still influence nonadherence. Overall, this finding is consistent with numerous studies showing that medical costs impede access to health care services across the cancer care continuum, including detection.^15,34,35,36,37^ The inability to afford preventive health care services due to cost both limits access but also perpetuates a cycle of worsening health outcomes.^38^ Although the 2010 Affordable Care Act mandated private insurance coverage for evidence-based cancer screenings without patient cost-sharing, in practice this does not always occur. For example, some insurance companies have reclassified procedures such as colonoscopies as diagnostic rather than screening tests if polyps are removed, leading to unexpected cost-sharing for patients.^39^ If a positive result on a noncolonoscopy screening test, such as a blood-stool test (promoted to increase CRCS rates in some socioeconomic and racial and ethnic subgroups) necessitates a follow-up colonoscopy, the follow-up procedure is often considered diagnostic, incurring further out-of-pocket costs for the patient.^39,40^ These billing practices, coupled with inconsistent pricing and aggressive collection tactics, can deter individuals from completing necessary screenings.^41^ Moreover, recent work has demonstrated a strong connection between medical debt and economic instability (eg, food insecurity, housing instability), emphasizing the broader implications of cost-related barriers to health care.^42^ Policy efforts focused on improving comprehensive insurance coverage and cost transparency may help reduce these barriers and support screening adherence.^43^ Although numerous interventions have focused on financial barriers, community-level advocacy, such as partnerships with insurers to lower screening costs, remains underused.^13,44,45^ Health systems should routinely screen patients for economic insecurities, and when needs are identified, they should facilitate and document connections to relevant community resources, including information on how needs were addressed. Prioritizing such initiatives could mitigate social risks, improve health care access, and foster equitable cancer prevention strategies. Moreover, rigorous research evaluating the effectiveness of health care system strategies and interventions to address social needs are urgently needed to inform evidence-based practices.

Transportation insecurity is a well-documented barrier to health care access and can arise from several factors.^46,47^ These include challenges with private transportation (eg, lack of vehicle access, inability to afford gas or parking), public transportation (infrastructure or payment), and/or time spent traveling to medical facilities.^46,48,49^ Our findings highlight the significant role transportation barriers play in cancer screening nonadherence, particularly for CRCS in women and BCS, where higher risk was observed. Our analyses also probed potential effect modification by sex and revealed heterogeneity in the magnitude and direction of some effect sizes. For example, upon stratification by sex for CRCS, the association persisted among women but not men. Additionally, while transportation insecurity corresponded with nonadherence for all screening types, an inverse effect was observed for LCS among men. Findings may indicate that gender-specific factors (eg, caregiving responsibilities, differential access to transportation resources) play a role in influencing adherence behaviors. Moreover, the varied impact of transportation insecurity on different screening modalities suggests the need for tailored interventions. Evidence indicates that interventions to address transportation to appointments were associated with the greatest improvement on screening rates^13^ and can produce health-system cost savings by decreasing no-show rates.^50^ Additionally, due to the unequal geographic distribution of medical facilities, mobile screening vans have emerged as an intervention to increase access among medically underserved communities.

Last, our findings highlight how social and community contextual factors (ie, life satisfaction, social and emotional support, social isolation, and mental distress) can impact adherence to cancer screening. Notably, life dissatisfaction was the only variable within this domain, where effects were in the same direction across all 4 screening types, particularly pronounced for BCS. Previous literature has established a strong correlation between life dissatisfaction and depressive symptoms^51^ and found that unmet mental health needs can deter participation in cancer screening.^21,52,53,54,55^ Therefore, integrating psychosocial considerations into interventions may be crucial for enhancing adherence, especially among women eligible for BCS. Among other factors in the social contextual domain, some cancer screening types suggested inverse effects, highlighting the variability in how these factors may relate to nonadherence. The explanation for these unexpected findings is not readily apparent and may be spurious but they warrant further investigation. One possible explanation may reflect the unique screening populations involved. For example, literature links the experience of systemic injustice to increased tobacco use.^56^ LCS targets individuals with past or present long-term cigarette smoking, unlike populations for other routine cancer screenings that generally consist of healthy individuals who qualify based on age and biological sex. These results indicate that while social support and mental health factors can impede adherence, their impact may vary significantly based on specific screening types. Research has also suggested that recent serious psychological distress may be a strong correlate of life dissatisfaction,^57^ which may indicate a more intricate association requiring complex analytical approaches to fully elucidate the underlying dynamics. Furthermore, qualitative research is essential for identifying specific barriers and facilitators associated with social and emotional support in this context. Longitudinal studies are also needed to assess how enhancements in support over time may influence screening adherence, providing insights for targeted interventions.^2^

### Limitations

Several key limitations should be considered when interpreting this study’s results. First, self-reported variables may be more susceptible to misclassification than other data collection methods. Notably, the wording of the CCS question changed in 2022, which could lead to underreporting, potentially affecting the accuracy and reliability of our results. Nevertheless, BRFSS data have been shown to be valid and reliable compared with other self-reported data, and self-reporting may be beneficial for key study variables such as race and ethnicity and perception of social risk. Second, although observational data can be susceptible to selection bias,^58^ BRFSS mitigates this through its weighting methodology, which accounts for the probability of study selection and adjusts for nonresponse. Nonetheless, the proportion of missing data for the SDOH-HE module was relatively high. This phenomenon may be attributed to the inclusion of cell phones in the survey design. Some respondents may have relocated to different states, and since optional modules (eg, SDOH-HE) are state specific, out-of-state cell phone respondents only complete the core module questions, leaving the optional modules incomplete. Third, the exclusion of certain vulnerable populations (eg, unhoused and institutionalized) limits the generalizability of the findings to groups who may experience heightened social risks. Furthermore, our findings may not be transportable to the populations outside the 39 states included in our study (ie, populations distinct from the study sample). Last, as with any observational study, we cannot completely rule out unmeasured confounding (eg, immigration status, medical mistrust, and/or discrimination).

## Conclusions

This cross-sectional study of social risks and nonadherence to guideline-recommended cancer screening revealed variations by screening test and sex. The associations between cost barriers and nonadherence, for instance, emphasize the need for health policy reforms to ensure access to screenings. To improve access, clinicians should assess patients’ social risks and connect them with community resources. Further research targeting specific populations is essential before effective interventions can be implemented, as social risks may not always align with patient-centered social needs.

## Notes

Supplement 1. **eFigure 1.** US States Administering Behavioral Risk Factor Surveillance System (BRFSS) Social Determinants of Health and Health Equity (SDOH-HE) Module in 2022 **eFigure 2.** Sample Size Selection **eTable 1.** Definitions of Screening Adherence for Colorectal, Lung, Cervical, and Breast Cancer, Based on the US Preventive Services Task Force (USPSTF) Recommendations **eTable 2.** Number of Respondents Excluded, by Screening Cohort **eTable 3.** Social Risk Measures—BRFSS, 2022 **eTable 4.** Prevalence of Social Risks Among Cancer-Screening-Eligible BRFSS Participants in 39 States and Washington, DC, 2022 **eTable 5.** Sensitivity Analysis—Odds Ratios and 95% CIs **eTable 6.** Sensitivity Analysis Exploring the Association of Social Risks With Colorectal Cancer Screening Guideline Nonadherence, by Gender **eTable 7.** Sensitivity Analysis Exploring the Association of Social Risks With Lung Cancer Screening Guideline Nonadherence, by Gender **eTable 8.** *P* Values for Table 3

Supplement 2.
Data Sharing Statement
