# Supplementary material for: Social Risks and Nonadherence to Recommended Cancer Screening Among US Adults
Source: JAMA Netw Open. 2025 Jan 3;8(1):e2449556. doi: 10.1001/jamanetworkopen.2024.49556 (PMC11699527; doi:10.1001/jamanetworkopen.2024.49556)
Supplement: Supplement 1. — eFigure 1. US States Administering Behavioral Risk Factor Surveillance System (BRFSS) Social Determinants of Health and Health Equity (SDOH-HE) Module in 2022 eFigure 2. Sample Size Selection eTable 1. Definitions of Screening Adherence for Colorectal, Lung, Cervical, and Breast Cancer, Based on the US Preventive Services Task Force (USPSTF) Recommendations eTable 2. Number of Respondents Excluded, by Screening Cohort eTable 3. Social Risk Measures—BRFSS, 2022 eTable 4. Prevalence of Social Risks Among Cancer-Screening-Eligible BRFSS Participants in 39 States and Washington, DC, 2022 eTable 5. Sensitivity Analysis—Odds Ratios and 95% CIs eTable 6. Sensitivity Analysis Exploring the Association of Social Risks With Colorectal Cancer Screening Guideline Nonadherence, by Gender eTable 7. Sensitivity Analysis Exploring the Association of Social Risks With Lung Cancer Screening Guideline Nonadherence, by Gender eTable 8. P Values for Table 3 [file jamanetwopen-e2449556-s001.pdf]

Supplementary Online Content

Sedani AE, Gomez SL, Lawrence WR, Moore JX, Brandt HM, Rogers CR. Social risks and nonadherence to recommended cancer screening among US adults. *JAMA Netw Open*. 2024;7(12):e2449556. doi:10.1001/jamanetworkopen.2024.49556

eFigure 1. US States Administering Behavioral Risk Factor Surveillance System (BRFSS) Social Determinants of Health and Health Equity (SDOH-HE) Module in 2022

eFigure 2. Sample Size Selection

eTable 1. Definitions of Screening Adherence for Colorectal, Lung, Cervical, and Breast Cancer, Based on the US Preventive Services Task Force (USPSTF) Recommendations

eTable 2. Number of Respondents Excluded, by Screening Cohort

eTable 3. Social Risk Measures—BRFSS, 2022

eTable 4. Prevalence of Social Risks Among Cancer-Screening-Eligible BRFSS Participants in 39 States and Washington, DC, 2022

eTable 5. Sensitivity Analysis—Odds Ratios and 95% CIs

eTable 6. Sensitivity Analysis Exploring the Association of Social Risks With Colorectal Cancer Screening Guideline Nonadherence, by Gender

eTable 7. Sensitivity Analysis Exploring the Association of Social Risks With Lung Cancer Screening Guideline Nonadherence, by Gender

eTable 8. *P* Values for Table 3

This supplementary material has been provided by the authors to give readers additional information about their work.

© 2024 Sedani AE et al. *JAMA Network Open*.

eFigure 1. US States Administering Behavioral Risk Factor Surveillance System (BRFSS) Social Determinants of Health and Health Equity (SDOH-HE) Module in 2022

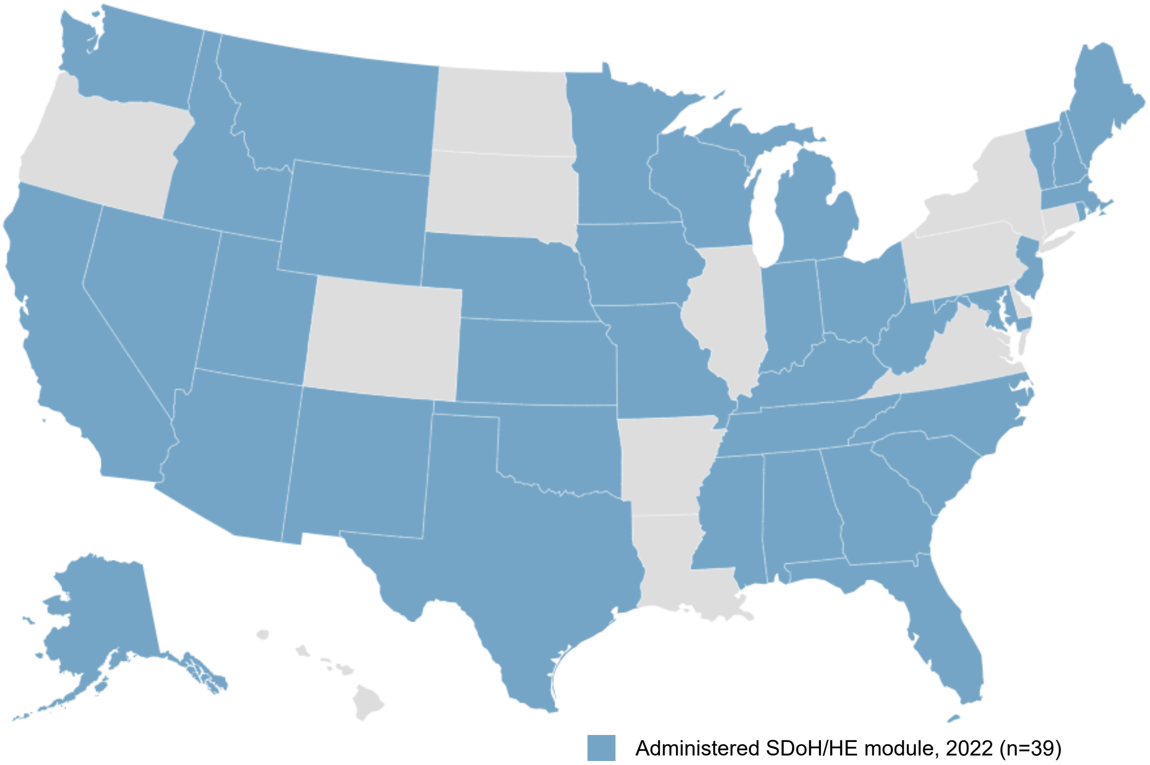

© 2024 Sedani AE et al. *JAMA Network Open*.

eFigure 2. Sample Size Selection

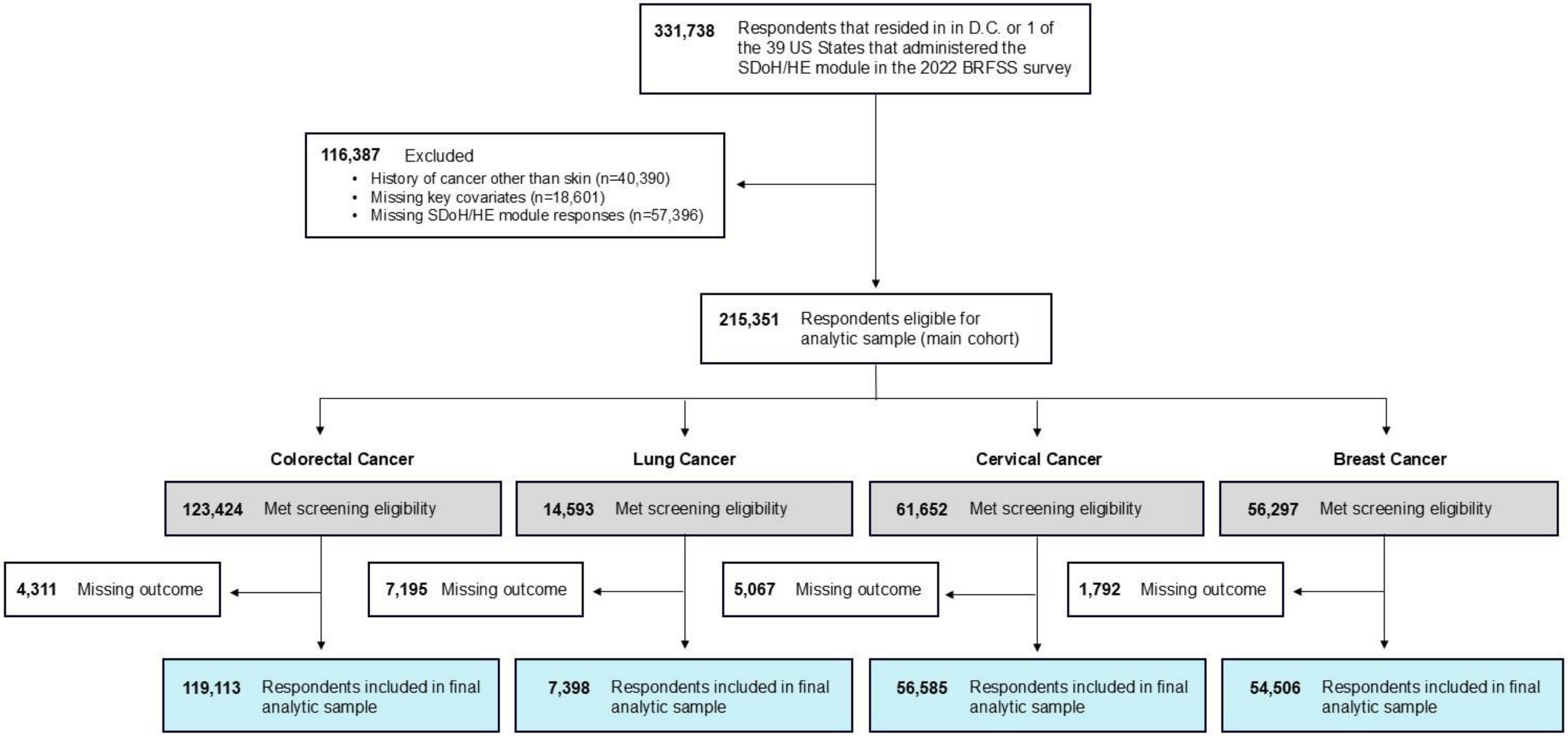

Note: number is unweighted count.

© 2024 Sedani AE et al. *JAMA Network Open*.

eTable 1. Definitions of Screening Adherence for Colorectal, Lung, Cervical, and Breast Cancer, Based on the US Preventive Services Task Force (USPSTF) Recommendations

**Cancer Type (Most Recent Update in Effect in 2022)**	**Cohort Eligibility**	**Adherence Definition**
**Colorectal** (2021)	• Aged 45–75 years	Completing at least 1 of the following tests:<br>• Blood stool test within the past year<br>• Sigmoidoscopy within the past 5 years<br>• Colonoscopy within the past 10 years<br>• Stool DNA test within the past 3 years<br>• Virtual colonoscopy within the past 5 years<br>• Sigmoidoscopy within the past 10 years ***and*** blood stool test in the past year
**Lung** (2021)	• Aged 50–80 years<br>• 20 pack-year cigarette smoking history<br>• Currently smoke cigarettes or quit within the past 15 years	• Low-dose CT within the past year
**Breast** (2016)	• Female respondent<br>• Aged 50–74 years	• Mammogram within the past 2 years
**Cervical** (2018)	• Female respondent<br>• Aged 21–65 years<br>• Have not had a hysterectomy*	Ages 21–29 years:<br>• Pap test (i.e., Pap smear, Cervical cytology) within the past 3 years
		Ages 30–65 years, at least 1 of the following:<br>• Pap test within the past 3 years<br>• hrHPV test within the past 5 years<br>• hrHPV testing in combination with Pap test (co-testing) within the past 5 years

Recommendations are for average-risk individuals; therefore, participants with a history of cancer were excluded from all cohorts.
Abbreviation: BRFSS, Behavioral Risk Factor Surveillance System; CT, computed tomography; hrHPV, high-risk human papilloma virus.
*Respondents who reported being pregnant at the time of survey administration were recoded as not having a hysterectomy.

© 2024 Sedani AE et al. *JAMA Network Open*.

eTable 2. Number of Respondents Excluded, by Screening Cohort

	No. (weighted %)			
	**Colorectal**	**Lung**	**Cervical**	**Breast**
**Ever had cancer**	25851 (12.1)	3324 (13.9)	5110 (5.0)	12881 (13.9)
**Covariates**	9931 (6.0)	1207 (5.7)	3515 (5.0)	4543 (6.0)
Education	1224 (0.8)	89 (0.4)	281 (0.3)	514 (0.7)
Martial	2395 (1.4)	157 (0.7)	721 (0.8)	1131 (1.4)
Urban/Rural	66 (0.004)	8 (0.006)	31 (0.006)	30 (0.005)
Health Insurance Coverage	6435 (3.9)	860 (4.0)	2282 (3.3)	3022 (4.0)
Personal Healthcare Provider	1524 (1.0)	202 (1.0)	581 (1.0)	590 (0.9)
**Exposure: SDoH**	38813 (26.3)	3821 (21.5)	12505 (21.2)	16661 (24.9)
Life Dissatisfaction	30965 (21.5)	2548 (15.2)	9676 (16.9)	13674 (20.6)
Lack of support	31987 (22.1)	2675 (15.5)	9611 (17.0)	13711 (20.9)
Feeling Isolated	31334 (21.8)	2556 (15.4)	9684 (17.2)	13740 (20.9)
Mentally Distressed	32123 (22.3)	2601 (15.8)	10200 (17.8)	14041 (21.2)
Employment Insecurity	31129 (21.7)	2501 (15.1)	9722 (17.2)	13751 (20.8)
Receiving SNAP	30659 (21.4)	2378 (14.5)	9668 (17.2)	13503 (20.6)
Food Insecurity	31225 (21.8)	2515 (15.1)	9769 (17.2)	13680 (20.7)
Housing Insecurity	31221 (21.8)	2469 (14.8)	9857 (17.4)	13710 (20.8)
Utility Services Insecurity	31187 (21.7)	2464 (14.9)	9847 (17.4)	13660 (20.7)
Transportation Insecurity	31291 (21.8)	2493 (15.1)	9911 (17.6)	13727 (20.7)
Cost as Barrier to care	653 (0.4)	105 (0.4)	228 (0.4)	264 (0.5)
**Outcome**	20496 (12.7)	10654 (49.2)	29800 (43.7)	9220 (12.5)
**Total unique excluded**	**69011**	**14504**	**41276**	**31325**

© 2024 Sedani AE et al. *JAMA Network Open*.

eTable 3. Social Risk Measures—BRFSS, 2022

SDOH Category	Item	BRFSS Question	Response Options	Recoding
**SOCIAL CONTEXT**	Life satisfaction	In general, how satisfied are you with your life? Are you…	1=Very satisfied<br>2= Satisfied<br>3= Dissatisfied<br>4= Very dissatisfied	1=Dissatisfied/Very dissatisfied<br>0=Satisfied/Very satisfied
	Social and emotional support	How often do you get the social and emotional support that you need? Is that…	1= Always<br>2= Usually<br>3= Sometimes<br>4= Rarely<br>5= Never	1=Sometimes/Rarely/Never<br>0=Always/Usually
	Social isolation	How often do you feel socially isolated from others? Is it…	1= Always<br>2= Usually<br>3= Sometimes<br>4= Rarely<br>5= Never	1=Always/Usually/Sometimes<br>0=Rarely/Never
	Mental distress	Stress means a situation in which a person feels tense, restless, nervous or anxious or is unable to sleep at night because their mind is troubled all the time. Within the last 30 days, how often have you felt this kind of stress? Was it…	1= Always<br>2= Usually<br>3= Sometimes<br>4= Rarely<br>5= Never	1=Always/Usually<br>0=Sometimes/Rarely/Never
**ECONOMIC STABILITY**	Lost or reduced hours for employment	In the past 12 months have you lost employment or had hours reduced?	1= Yes<br>2= No	1=Yes<br>0=No
	Receiving food stamps or SNAP	During the past 12 months, have you received food stamps, also called SNAP, the Supplemental Nutrition Assistance Program on an EBT card?	1= Yes<br>2= No	1=Yes<br>0=No
	Food insecurity	During the past 12 months how often did the food that you bought not last, and you didn't have money to get more? Was that…	1= Always<br>2= Usually<br>3= Sometimes<br>4= Rarely	1=Always/Usually/Sometimes<br>0=Rarely/Never

© 2024 Sedani AE et al. *JAMA Network Open*.

			5= Never	
	Housing insecurity	During the last 12 months, was there a time when you were not able to pay your mortgage, rent or utility bills?	1= Yes<br>2= No	1=Yes<br>0=No
	Threatened to shut off utility services	During the last 12 months was there a time when an electric, gas, oil, or water company threatened to shut off services?	1= Yes<br>2= No	1=Yes<br>0=No
**BUILT ENVIRONMENT**	Lack of reliable transportation	During the past 12 months has a lack of reliable transportation kept you from medical appointments, meetings, work, or from getting things needed for daily living?	1= Yes<br>2= No	1=Yes<br>0=No
**HEALTH AND HEALTH CARE**	Barrier as a cost to care	Was there a time in the past 12 months when you needed to see a doctor but could not because you could not afford it?	1= Yes<br>2= No	1=Yes<br>0=No

© 2024 Sedani AE et al. *JAMA Network Open*.

eTable 4. Prevalence of Social Risks Among Cancer-Screening-Eligible BRFSS Participants in 39 States and Washington, DC, 2022

Social Risks	Respondents, No. (weighted % [95% CI])			
	**Adults Eligible for CRCS** (n = 119,113)	**Adults Eligible for LCS** (n =7,398)	**Adults Eligible for CCS** (n = 56,585)	**Adults Eligible for BCS** (n =54,506)
**Life Dissatisfaction**				
No	113,302 (95.1 [94.8- 95.3])	6568 (87.9 [86.5- 89.3]	53,530 (94.2 [93.8- 94.6])	51,726 (94.8 [94.4- 95.2])
Yes	5,811 (4.9 [4.7- 5.2])	830 (12.1 [10.7- 13.5]	3,055 (5.8 [5.4- 6.2])	2,780 (5.2 [4.8- 5.6])
**Lack of Support**				
No	96,154 (78.8 [78.3- 79.3])	5125 (68.4 [66.4- 70.5]	44,914 (76.9 [76.1- 77.6])	44,664 (80.1 [79.3- 80.9])
Yes	22,959 (21.2 [20.7- 21.8])	2273 (31.6 [29.5- 33.6]	11,671 (23.1 [22.4- 23.9])	9,842 (19.9 [19.1- 20.7])
**Feeling Isolated**				
No	87,771 (74.1 [73.6- 74.7])	4662 (63.9 [61.9- 65.9]	38,366 (66.6 [65. 8- 67.4])	39,283 (72.5 [71.7- 73.4])
Yes	31,342 (25.9 [25.3- 26.4])	2736 (36.1 [34.1- 38.1]	18,219 (33.4 [32.6- 34.2])	15,223 (27.5 [26.6- 28.3])
**Mentally Distressed**				
No	107,936 (90.0 [89.6- 90.4])	6044 (81.3 [79.6- 83]	47,337 (82.7 [82.0- 83.3])	48,873 (89.0 [88.4- 89.6])
Yes	11,177 (10.0 [9.6- 10.4])	1354 (18.7 [17- 20.4]	9,248 (17.4 [16.7- 18.0])	5,633 (11.0 [10.5- 11.6])
**Employment Instability**				
No	110,050 (90.5 [90.1- 90.9])	6733 (89.5 [88.2- 90.8]	49,647 (85.0 [84.3- 85.7])	50,695 (91.7 [91.0- 92.4])
Yes	9,063 (9.5 [9.1- 9.9])	665 (10.5 [9.2- 11.8]	6,938 (15.0 [14.3- 15.7])	3,811 (8.4 [7.7- 9.0])
**Receiving SNAP**				
No	108,933 (90.4 [90.0- 90.8])	5573 (73.2 [71.2- 75.2]	48,721 (82.6 [81.9- 83.3])	49,178 (89.1 [88.4- 89.7])
Yes	10,180 (9.6 [9.2- 10.0])	1825 (26.8 [24.8- 28.8]	7,864 (17.4 [16.7- 18.1])	5,328 (10.9 [10.3- 11.6])
**Food Insecurity**				
No	108,098 (88.1 [87.7- 88.5])	5692 (73.9 [72- 75.8]	49,146 (83.4 [82.7- 84.0])	48,982 (86.6 [85.9- 87.3])
Yes	11,015 (11.9 [11.5- 12.3])	1706 (26.1 [24.2- 28]	7,439 (16.6 [16.0- 17.3])	5,524 (13.4 [12.7- 14.1])
**Housing Insecurity**				
No	110,114 (90.7 [90.4- 91.1])	6086 (80.5 [78.8- 82.3]	49,097 (84.1 [83.5- 84.7])	50,215 (90.4 [89.8- 91.0])
Yes	8,999 (9.3 [8.9- 9.7])	1312 (19.5 [17.7- 21.2]	7,488 (15.9 [15.3- 16.6])	4,291 (9.6 [9.0- 10.2])

© 2024 Sedani AE et al. *JAMA Network Open*.

**Utility Services Insecurity**				
No	112,867 (93.8 [93.4- 94.1])	6495 (85.8 [84.2- 87.3]	51,668 (89.9 [89.3- 90.4])	51,628 (93.3 [92.7- 93.8])
Yes	6,246 (6.3 [5.9- 6.6])	903 (14.2 [12.7- 15.8]	4,917 (10.1 [9.6- 10.7])	2,878 (6.7 [6.2- 7.3])
**Transportation Instability**				
No	113,049 (94.4 [94.1- 94.7])	6379 (85.6 [84.1- 87.1]	52,258 (91.2 [90.8, 91.8])	51,499 (94.1 [93.7- 94.5])
Yes	6,064 (5.6 [5.3- 5.9])	1019 (14.4 [12.9- 15.9]	4,327 (8.8 [8.3, 9.2])	3,007 (5.9 [5.48- 6.3])
**Cost is Barrier to Care**				
No	111,391 (92.1 [91.8- 92.5])	6487 (85.9 [84.2- 87.5]	49,794 (85.4 [84.8, 86.1])	22,746 (90.9 [90.1- 91.6])
Yes	7,722 (7.9 [7.5- 8.2])	911 (14.1 [12.5- 15.8]	6,791 (14.6 [14.0, 15.2])	1,881 (9.1 [8.4- 9.9])

Abbreviations: CRCS, Colorectal Cancer Screening; LCS, Lung Cancer Screening; CCS, Cervical Cancer Screening; BCS, Breast Cancer Screening; SNAP, Supplemental Nutrition Assistance Program.

© 2024 Sedani AE et al. *JAMA Network Open*.

eTable 5. Sensitivity Analysis—Odds Ratios and 95% CIs

	**OR (95% CI)** [a]			
	**Colorectal**	**Lung**	**Cervical**	**Breast**
**SOCIAL CONTEXT**				
**Life Dissatisfaction**	1.14 (1.01-1.30)	0.78 (0.55- 1.12)	1.14 (0.90-1.43)	1.93 (1.62-2.28)
**Lack of Support**	1.22 (1.13-1.31)	1.05 (0.80- 1.38)	1.26 (1.09-1.46)	1.40 (1.24-1.58)
**Feeling Isolated**	1.12 (1.05-1.20)	1.11 (0.88- 1.40)	1.12 (0.99-1.27)	1.37 (1.24-1.51)
**Mentally Distressed**	1.04 (0.94-1.15)	0.85 (0.64- 1.13)	1.16 (1.00-1.34)	1.32 (1.16-1.50)
**ECONOMIC STABILITY**				
**Employment Insecurity**	1.17 (1.03-1.32)	0.88 (0.61- 1.27)	0.95 (0.76-1.19)	1.24 (1.04-1.49)
**Receiving Food Stamps (SNAP)**	1.21 (1.08-1.36)	1.00 (0.74- 1.34)	1.06 (0.85-1.31)	1.43 (1.21-1.69)
**Food Insecurity**	1.31 (1.18-1.46)	0.84 (0.64- 1.11)	1.36 (1.12-1.64)	1.53 (1.32-1.78)
**Housing Insecurity**	1.23 (1.10-1.38)	0.73 (0.54- 1.00)	1.16 (0.96-1.39)	1.61 (1.38-1.88)
**Utility Services Insecurity**	1.28 (1.12-1.46)	0.79 (0.56- 1.09)	1.19 (0.95-1.48)	1.85 (1.52-2.25)
**BUILT ENVIRONMENT**				
**Transportation Insecurity**	1.31 (1.16-1.49)	1.12 (0.78- 1.61)	1.33 (1.08-1.65)	1.89 (1.59-2.25)
**HEALTH AND HEALTH CARE**				
**Cost Is Barrier To care**	1.39 (1.23-1.56)	1.52 (1.08- 2.15)	1.21 (1.00-1.47)	2.04 (1.75-2.39)
**TOTAL ADVERSE SOCIAL RISKS**	1.07 (1.05-1.10)	1.04 (0.98- 1.10)	1.00 (0.98-1.02)	1.17 (1.13-1.20)

[a] **Adjusting for**: gender (CRC and LCS), age, race and ethnicity (proxy systemic racism), educational attainment, health insurance plan, marital status, and geography: census region (proxy access to services, policy, and cultural differences), accounting for complex survey design.

© 2024 Sedani AE et al. *JAMA Network Open*.

eTable 6. Sensitivity Analysis Exploring the Association of Social Risks With Colorectal Cancer Screening Guideline Nonadherence, by Gender

	**RR (95% CI)[a]**		
	**Total**	**Men** (n=54,935)	**Women** (n=64,178)
**SOCIAL CONTEXT**			
**Life dissatisfaction**	1.04 (1.00, 1.09)	1.05 (0.99, 1.12)	1.04 (0.98, 1.10)
**Lack of social & emotional support**	1.06 (1.04, 1.09)	1.08 (1.04, 1.12)	1.04 (1.01, 1.08)
**Feeling socially isolated**	1.03 (1.01, 1.05)	1.04 (1.01, 1.07)	1.03 (1.00, 1.05)
**Mentally distressed**	1.01 (0.98, 1.04)	1.00 (0.95, 1.05)	1.02 (0.98, 1.06)
Total Score	1.02 (1.01, 1.03)	1.03 (1.01. 1.05)	1.02 (1.00, 1.03)
**ECONOMIC STABILITY**			
**Employment insecurity**	1.06 (1.01, 1.11)	1.15 (1.08, 1.23)	1.07 (1.00, 1.14)
**Receiving food stamps (SNAP)**	1.06 (1.02, 1.11)	1.05 (0.99, 1.12)	1.08 (1.02, 1.14)
**Food insecurity**	1.10 (1.06, 1.14)	1.11 (1.04, 1.17)	1.09 (1.04, 1.15)
**Housing insecurity**	1.08 (1.04, 1.13)	1.17 (1.08, 1.25)	1.04 (0.98, 1.09)
**Utility services insecurity**	1.10 (1.04, 1.15)	1.18 (1.08, 1.29)	1.05 (0.99, 1.12)
Total Score	1.04 (1.03, 1.06)	1.06 (1.03, 1.08)	1.04 (1.02, 1.06)
**BUILT ENVIRONMENT**			
**Lack of reliable transportation**	1.11 (1.06, 1.16)	1.06 (0.98, 1.14)	1.15 (1.08, 1.22)
**HEALTH AND HEALTH CARE**			
**Cost is barrier to health care access**	1.14 (1.09, 1.20)	1.13 (1.05, 1.22)	1.15 (1.08, 1.23)
**Total adverse social risks**	1.03 (1.02, 1.03)	1.03 (1.02, 1.04)	1.02 (1.01, 1.03)

[a] **Adjusting for**: gender (total only), age, race and ethnicity (proxy systemic racism), educational attainment, health insurance coverage, marital status, and geography: census region (proxy access to services, policy, and cultural differences), accounting for complex survey design (weight).

© 2024 Sedani AE et al. *JAMA Network Open*.

eTable 7. Sensitivity Analysis Exploring the Association of Social Risks With Lung Cancer Screening Guideline Nonadherence, by Gender

	**RR (95% CI)** [a]		
	**Total** (n=7,398)	**Men** (n=3,678)	**Women** (n=3,720)
**SOCIAL CONTEXT**			
**Life dissatisfaction**	1.23 (0.91- 1.66)	1.18 (0.76- 1.83)	1.29 (0.88- 1.89)
**Lack of social & emotional support**	0.96 (0.76- 1.22)	0.84 (0.61- 1.16)	1.14 (0.87- 1.49)
**Feeling socially isolated**	0.92 (0.76- 1.12)	0.96 (0.72- 1.29)	0.88 (0.70- 1.12)
**Mentally distressed**	1.15 (0.90- 1.46)	1.15 (0.78- 1.68)	1.19 (0.89- 1.60)
Total Score	1.01 (0.93- 1.09)	0.98 (0.87- 1.11)	1.04 (0.94- 1.14)
**ECONOMIC STABILITY**			
**Employment insecurity**	1.12 (0.80- 1.56)	0.91 (0.61- 1.34)	1.55 (0.87- 2.77)
**Receiving food stamps (SNAP)**	1.00 (0.79- 1.28)	0.86 (0.62- 1.20)	1.24 (0.90- 1.71)
**Food insecurity**	1.15 (0.92- 1.44)	1.18 (0.85- 1.63)	1.12 (0.83- 1.50)
**Housing insecurity**	1.03 (0.99- 1.70)	1.19 (0.81- 1.76)	1.46 (1.03- 2.08)
**Utility services insecurity**	1.22 (0.92- 1.63)	1.24 (0.80- 1.92)	1.22 (0.84- 1.77)
Total Score	1.08 (0.98- 1.18)	1.03 (0.90- 1.18)	1.13 (1.01- 1.28)
**BUILT ENVIRONMENT**			
**Lack of reliable transportation**	0.92 (0.68- 1.24)	0.81 (0.53- 1.26)	1.10 (0.75- 1.60)
**HEALTH AND HEALTH CARE**			
**Cost is barrier to health care access**	1.43 (1.05- 1.93)	1.29 (0.84- 1.98)	1.54 (1.01- 2.33)
**Total adverse social risks**	1.03 (0.98- 1.08)	1.00 (0.94- 1.07)	1.06 (1.00- 1.13)

[a] **Adjusting for**: gender (total only), age, race and ethnicity (proxy systemic racism), educational attainment, health insurance coverage, marital status, and geography: census region (proxy access to services, policy, and cultural differences), accounting for complex survey design (weight).

© 2024 Sedani AE et al. *JAMA Network Open*.

eTable 8. *P* Values for Table 3

	Colorectal		Lung		Cervical	Breast
	Men	Women	Men	Women		
**SOCIAL CONTEXT**						
**Life Dissatisfaction**	0.12	0.19	0.46	0.19	0.03	<.001
**Lack of support**	<.001	0.01	0.30	0.35	0.22	<.001
**Feeling Isolated**	0.01	0.07	0.79	0.31	0.43	<.001
**Mentally Distressed**	0.91	0.41	0.48	0.25	0.07	<.001
Total Social Score	<.001	0.01	0.80	0.47	0.46	<.001
**ECONOMIC STABILITY**						
**Employment Insecurity**	0.09	0.05	0.62	0.14	0.09	0.03
**Receiving SNAP**	0.10	0.01	0.38	0.18	0.19	<.001
**Food Insecurity**	<.001	<.001	0.33	0.47	0.04	<.001
**Housing Insecurity**	<.001	0.19	0.38	0.03	0.03	<.001
**Utility Services Insecurity**	<.001	0.13	0.34	0.29	0.07	<.001
Total Economic Score	<.001	<.001	0.65	0.04	0.14	<.001
**BUILT ENVIRONMENT**						
**Transportation Insecurity**	0.15	<.001	0.35	0.62	0.07	<.001
**HEALTH AND HEALTH CARE**						
**Cost is barrier to care**	<.001	<.001	0.25	0.04	0.26	<.001
**Total adverse social risks**	<.001	<.001	0.91	0.06	0.99	<.001

© 2024 Sedani AE et al. *JAMA Network Open*.
