# Supplementary material for: Social Risks and Nonadherence to Recommended Cancer Screening Among US Adults
Source: JAMA Netw Open. 2025 Jan 3;8(1):e2449556. doi: 10.1001/jamanetworkopen.2024.49556 (PMC11699527; doi:10.1001/jamanetworkopen.2024.49556)
Supplement: Supplement 2. — Data Sharing Statement [file jamanetwopen-e2449556-s002.pdf]

Data Sharing Statement

Sedani. Social Risks and Nonadherence to Recommended Cancer Screening Among US Adults. *JAMA Netw Open*. Published December 13, 2024.
doi:10.1001/jamanetworkopen.2024.49556

Data
Data available: No

Additional Information
Explanation for why data not available: This is a publicly available dataset. The data dictionary is so available online.
